# The impact of PRDX4 and the EGFR mutation status on cellular proliferation in lung adenocarcinoma

**DOI:** 10.7150/ijms.36071

**Published:** 2019-08-14

**Authors:** Kenichi Mizutani, Xin Guo, Akihiro Shioya, Jing Zhang, Jianbo Zheng, Nozomu Kurose, Hiroaki Ishibashi, Nozomu Motono, Hidetaka Uramoto, Sohsuke Yamada

**Affiliations:** 1 Departments of Pathology and Laboratory Medicine, Kanazawa Medical University, Ishikawa, Japan.; 2 Departments of Oral and Maxillofacial Surgery, Kanazawa Medical University, Ishikawa, Japan.; 3 Departments of Thoracic Surgery, Kanazawa Medical University, Ishikawa, Japan.

**Keywords:** PRDX4, EGFR, cell proliferation, prognosis, lung adenocarcinoma (LUAD).

## Abstract

**Background:** Oxidative stress plays key roles in the progression of lung adenocarcinoma. Recently, we reported that peroxiredoxin 4 (PRDX4), an antioxidant enzyme, can be a prognostic marker of lung adenocarcinoma (LUAD). In the present study, we aimed to further investigate the relationship among the PRDX4 expression, epidermal growth factor receptor (EGFR) mutations and cell proliferation in LUAD.

**Methods:** The expression of PRDX4 was immunohistochemically analyzed and the EGFR mutation status was examined in 127 paraffin-embedded human surgical specimens from patients with stage I LUAD. The PRDX4 expression was considered to be high when >40% of the adenocarcinoma cells were positively stained. *In vitro*, using plasmid transfection methods, PRDX4 plasmid DNAs were transfected into human lung adenocarcinoma cell lines, A549 (EGFR-wild) or PC-9 (EGFR mutant). The viability of these cells was analyzed using a Cell Counting Kit-8 kit.

**Results:** The number of cases with high PRDX4 expression levels among patients with LUAD with EGFR mutations was significantly larger than that in patients with EGFR wild-type. The combination of the PRDX4 expression level with the EGFR mutation status was closely associated with the prognosis of patients with stage I LUAD. Viability assays showed that the proliferation of A549 cells was significantly suppressed after PRDX4 plasmid transfection, while the overexpression of PRDX4 had no effect on the proliferation of EGFR-mutant PC-9 cells.

**Conclusions:** The PRDX4 expression and EGFR mutation status were significantly associated with the prognosis of patients with stage I LUAD, and EGFR mutations affected the role of PRDX4 in the proliferation of LUAD cells.

## Introduction

Lung cancer has been one of the leading causes of cancer-related death in the world for two decades 1. There were up to 370,000 cancer deaths and more than 74,000 lung cancer deaths in Japan in 2017. More than half of lung cancer cases are classified as non-small cell lung cancer (NSCLC); lung adenocarcinoma (LUAD) is a predominant subtype of these cases 2. The 5-year overall survival rate is considered to be <20% for NSCLC 3. Amazingly, up to 30% of patients develop recurrent disease within 5 years, even those with stage Ⅰ LUAD 4.

Oxidative stress can function as a crucial and diverse pathophysiological regulator of cellular signaling pathways, including growth factor stimulation 5, which can lead to the development of malignant neoplasms. In LUAD, the excessive expression of oxidative stressors plays a pivotal role in progression through cell signaling pathways closely related to tumor growth 6, 7. Peroxiredoxins (PRDXs), a new family of antioxidant enzymes, including at least six distinct PRDX genes expressed in mammals (PRDX1-6) 8, are ubiquitously synthesized and abundantly identified in various organisms. Recently, a large amount of clinical evidence indicates that PRDXs play important roles in the malignant progression of many cancers 9-13.

PRDX4, an antioxidant enzyme, is the only known secretory form of the PRDX family and is uniquely located in both the intracellular space and the extracellular space 14, 15. In our previous series of studies, we showed that the overexpression of PRDX4 could prevent the progression of metabolic syndrome by reducing local and systemic oxidative stress and suppressing steatosis, inflammatory reactions, and/or apoptotic activity, suggesting that PRDX4 might be useful in the treatment of various chronic inflammatory diseases 16-19. Recently, evidence demonstrated that different PRDX4 expression levels in tumor tissues were closely associated with the prognosis of cancer patients, indicating this antioxidant enzyme played important roles in the initiation and progression of cancer 20, 21. In lung cancer, a report showed that high PRDX4 expression levels were significantly correlated with higher rates of recurrence and shorter disease-free survival (DFS) in patients with lung squamous cell carcinoma, but not in patients with lung adenocarcinoma 22. Most recently, we reported that human LUAD tissues with low PRDX4 expression levels, a highly malignant phenotype that is very closely related to poor differentiation, highly invasive characteristics and recurrence, and the combination of low-PRDX4 and a high Ki-67 (MIB-1) labelling index may be a novel and useful independent predictor of recurrence with a poor prognosis in patients with primary stage I LUAD 23.

The epidermal growth factor receptor (EGFR), penetrating the cell membrane, is a member of the extensively studied receptor tyrosine kinase family. It can be activated by a family of ligands and induces downstream signaling pathways, which plays important roles in cell proliferation, differentiation, migration, and survival 24, 25. Mutation of the EGFR gene leads to the aberrant activation of downstream signaling pathways and participates in critical mechanisms promoting tumorigenesis in malignant diseases, especially lung cancer 26, 27. Pathologically, the aberrant expression of EGFR was found in more than half of non-small-cell lung cancer (NSCLC) patients 28 and mutations in the kinase domain of the EGFR gene are detected in approximately 40% of East Asian lung adenocarcinoma patients 29. The EGFR mutation status has become one of the most important factors in the selection of lung cancer treatment, since EGFR Tyr kinase inhibitors (TKIs) have been widely used to treat NSCLC patients in recent years 30. However, highly metastatic properties and drug resistance during TKI therapy—the precise mechanisms of which remain unclear—are still a problem to be solved for these NSCLC patients. It is well known that reactive oxygen species (ROS) are heavily involved in cancer initiation and regulation 31, and oxidative stress formation is an important mechanism participating in lung tumorigenesis through the regulation of the EGFR-mediated signaling pathway 32, 33.

One study reported that the intracellular overexpression of mouse PRDX4 prevented the production of ROS induced by epidermal growth factor (EGF) 34, indicating that EGFR may play an important role in the signaling pathways. Thus, there may be a close relationship between the expression of PRDX4 and EGFR mutation. However, there have been no studies on the relationships among the PRDX4 expression, the EGFR mutation status and cellular proliferation in LUAD. In this study, we examined the PRDX4 expression and EGFR mutation status in surgical specimens from patients with stage I LUAD. We also investigated the different roles of PRDX4 in the proliferation of EGFR wild-type and EGFR mutants in human LUAD cell lines *in vitro*.

## Materials and methods

### Patients

In the present study, we evaluated surgically resected stage I LUAD tissue specimens in which the EGFR mutation status had been evaluated. All specimens were obtained from patients who were treated at Kanazawa Medical University from January 2005 to December 2015. All materials in this article were approved by the Ethical Committee of Kanazawa Medical University (I159). Patients who suffered perioperative death, defined as death during the patient's initial hospitalization or within 30 days of surgery, were excluded. In addition, patients with the following characteristics were excluded: (a) other prior or concomitant malignant tumours, (b) coexisting medical problems of sufficient severity to shorten the life expectancy, and (c) adjuvant chemotherapies or radiotherapies prior to the surgery. After applying the exclusion criteria, a total of 127 patients with available follow-up data were included in this retrospective study.

Formalin-fixed, paraffin-embedded specimens were used for the IHC study. Clinical information was gathered from patient records. Patients were followed for 5 years after surgery. Disease-free survival (DFS) and disease-specific survival (DSS) were defined as the interval from the date of surgery to recurrence and from the date of surgery to death (except for patients who died from causes other than LUAD) or the most recent clinic visit, respectively.

### Histology and immunohistochemistry of tissue samples

Three certified pathologists examined all resected specimens to evaluate their histopathological features. Tumors were classified according to the International Association for the Study of Lung Cancer (IASLC)/American Thoracic Society (ATS)/European Respiratory Society (ERS) classification 35. A rabbit anti-PRDX4 IgG was produced and immunohistochemical staining was performed as previously described 23, 36. PRDX4 immunoreactivity was determined semi-quantitatively by evaluating the proportion of positively stained cells in comparison to the total number of adenocarcinoma cells.

### Cell culture

A549, a LUAD cell line with wild-type EGFR, was obtained from the Department of Pathology, Kagoshima University. PC-9, a human LUAD cell line with EGFR mutation, was purchased from Riken BioResource Center (Tsukuba, Japan). These cells were cultured in Dulbecco's Modified Eagle Medium (DMEM) with 10% fetal calf serum and were maintained in a humidified atmosphere at 37°C under 95% air/5% CO_2_.

### Cell transfection

PRDX4 plasmid DNA was obtained from the Department of Pathology, Kagoshima University. The cells were plated in 6-well plates and cultured in growth media at approximately 60% confluence, incubated for 24 h, and transfected for 72 h with 5 µg PRDX4 plasmid DNA duplexes in Lipofectamine^®^ 2000 and Opti-MEM medium (Life Technologies, Carlsbad, CA, USA). PMCV-Tag-2b (a gift from the Department of Pathology, Kagoshima University) vectors were used as a negative control respectively.

### Real-time PCR

Total RNA from PC-9 and A549 cells was extracted using a Relia Prep RNA Cell Miniprep System (Promega) and was converted into cDNA using a High Capacity RNA-to-cDNA Kit (applied biosystems). The cDNA was analyzed using an Applied Biosytems QuantStudio 12K Flex Real-Time PCR system (Life Technologies) with TaqMan gene expression assays (Life Technologies). Each sample was analyzed in triplicate with separate wells for PRDX4 and ribosomal 18S genes. The average values of three threshold cycle values for PRDX4 and 18S were calculated using the comparative Ct method. Custom-made primers and the TaqMan probe for PRDX4 gene amplification were purchased from Life Technologies (Assay ID: Hs01056076_m1).

### Western blotting

Proteins isolated from A549 and PC-9 cells were separated by sodium dodecyl sulfate/polyacrylamide gel electrophoresis and transferred to PVDF Western Blotting Membranes (Roche Diagnostics GmbH) using a semidry blotter. After transfer, the membranes were blocked with 5% skim milk in TBST (TBS and 0.1% Tween 20 solution) for 1 h at room temperature (RT) and then incubated at RT with primary antibody diluted in TBST. The following primary antibodies and dilutions were used: PRDX4 Antibody (1:1000; Invitrogen) (rabbit polyclonal antibody), Anti β-Actin, MoAb, Peroxidase Conjugated (1:1000; Wako) (rabbit monoclonal antibody). After incubation with the primary antibody for PRDX4, the membrane was incubated with Anti-rabbit IgG, HRP-linked Antibody (1:1000; Cell Signaling) for 1 h at RT. The protein expression was detected with Clarity Western ECL Substrate (Bio-Rad).

### Cell proliferation assay

The CCK-8 method was used to measure the viability of cells according to the manufacturer's instructions. A549 or PC-9 cells (1×10^3^ cells) were seeded in one well of a 96-well plate and were observed for 72 h. Absorbance at 450 nm was measured using a microplate reader.

### Statistical analyses

All statistical analyses were performed using the R software program (version 3.2.3). The χ^2^ test or Fisher's exact test was used to assess relationships among the immunohistochemical expression of PRDX4, the EGFR mutation status and the clinicopathological features. Survival curves were analyzed using the Kaplan-Meier method and were compared using log-rank tests. The *t-*test was used to analyze continuous variables. All statistical analyses were two sided. P values of <0.05 were considered to indicate statistical significance.

## Results

### Patient characteristics

The clinicopathological features of the 127 patients with stage I LUAD who were evaluated in the present study are summarized in Table 1. The age of the patients at surgery range from 34 to 84 years (average, 68 years; median, 69 years). More than half of the patients (80/127) had a Brinkman index (BI) of <400. The tumors were graded as follows: well-differentiated, n=72; moderately differentiated, n=48; and poorly differentiated, n=7. The tumor size ranged from 6 mm to 50 mm (average, 22; median, 21 mm). Pleural invasion, lymphatic invasion and venous invasion were found in 22, 46 and 43 cases, respectively.

### The PRDX4 expression level and EGFR mutation status were correlated with the prognosis of patients with stage I LUAD

**Figure 1:**
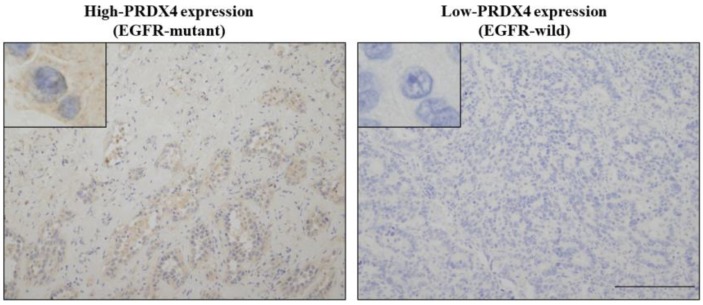
** Representative images of the immunohistochemical analysis of PRDX4 (left, high-PRDX4; right, low-PRDX4) in patients with EGFR wild-type or EGFR mutations.** The intracytoplasmic staining pattern of PRDX4 was confirmed. (Original magnification: ×100; inset, ×400). Bar = 200 μm (×100).

**Figure 2:**
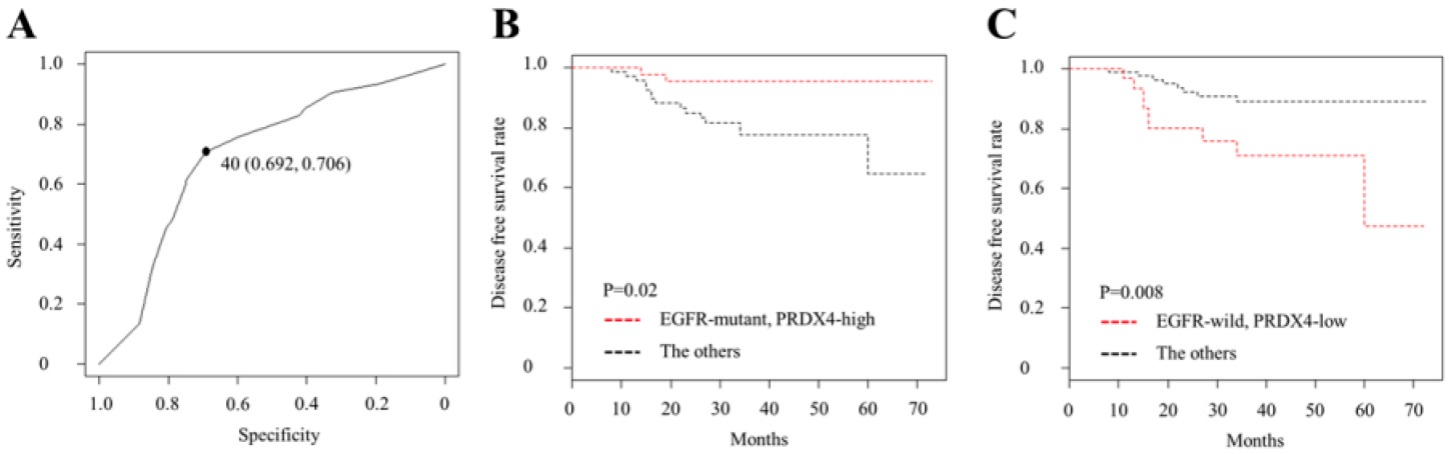
** The receiver operating characteristic (ROC) curve analysis and Kaplan-Meier curves of the disease-free survival (DFS) in patients with Stage I LUAD after surgery according to the PRDX4 expression and the EGFR mutation status. (A)** We selected 40% as the cut-off point for PRDX4, since the sum of sensitivity and specificity was the highest at this point. **(B)** Patients with EGFR mutations and high-PRDX4 showed significantly longer postsurgical DFS. **(C)** Patients with EGFR wild-type and low-PRDX4 showed significantly shorter postsurgical DFS.

To evaluate the relevance between the PRDX4 expression and EGFR mutation status and their clinical significance in stage I LUAD, we investigated the PRDX4 expression levels by IHC and examined the EGFR mutation status in 127 stage I LUAD tissue specimens. Based on the IHC staining scores, which were evaluated by three professional pathologists, the cases were divided into the low-PRDX4 and high-PRDX4 groups (Fig. 1) using a receiver operating characteristic (ROC) curve (Fig. 2A). The number of cases with EGFR gene mutations was compared between the two groups. Our results showed that the proportion of cases with EGFR gene mutations in the high-PRDX4 group (mutant, n=55; wild-type, n=16) was significantly higher than that in the low-PRDX4 group (mutant, n=22; wild-type, n=36), suggesting that the PRDX4 expression level was significantly correlated with the EGFR mutation status in stage I LUAD (Table 2). Furthermore, in a Kaplan-Meier analysis, patients with stage I LUAD, high-PRDX4 and an EGFR mutation had a significantly longer postoperative DFS than other patients (P = 0.02, Fig. 2B), while patients with stage I LUAD, low-PRDX4 and EGFR wild-type had significantly shorter postoperative DFS than other patients (P = 0.008, Fig. 2C), However, the PRDX4 expression and EGFR mutation status was not associated with the clinicopathological characteristics of the cohort in the present study (Supplementary Tables 1 and 2).

### The overexpression of PRDX4 inhibited the proliferation of LUAD cells, but did not affect the LUAD cells with EGFR mutations

**Figure 3:**
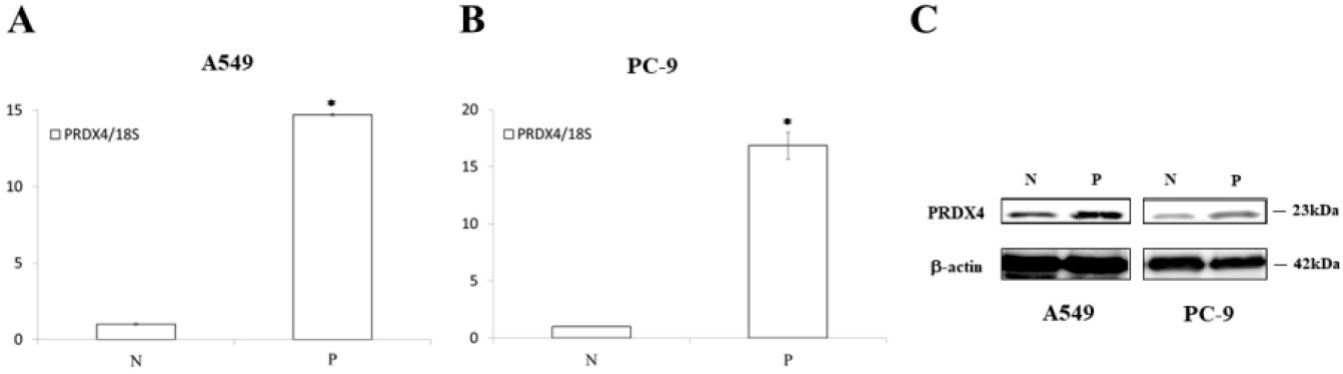
** The results of the real-time PCR and Western blotting.** The PRDX4 mRNA **(A, B)** and protein **(C)** expression was remarkably increased after transfection of PRDX4 plasmid in A549 and PC-9. N, negative control vector; P, PRDX4 plasmid.

**Figure 4:**
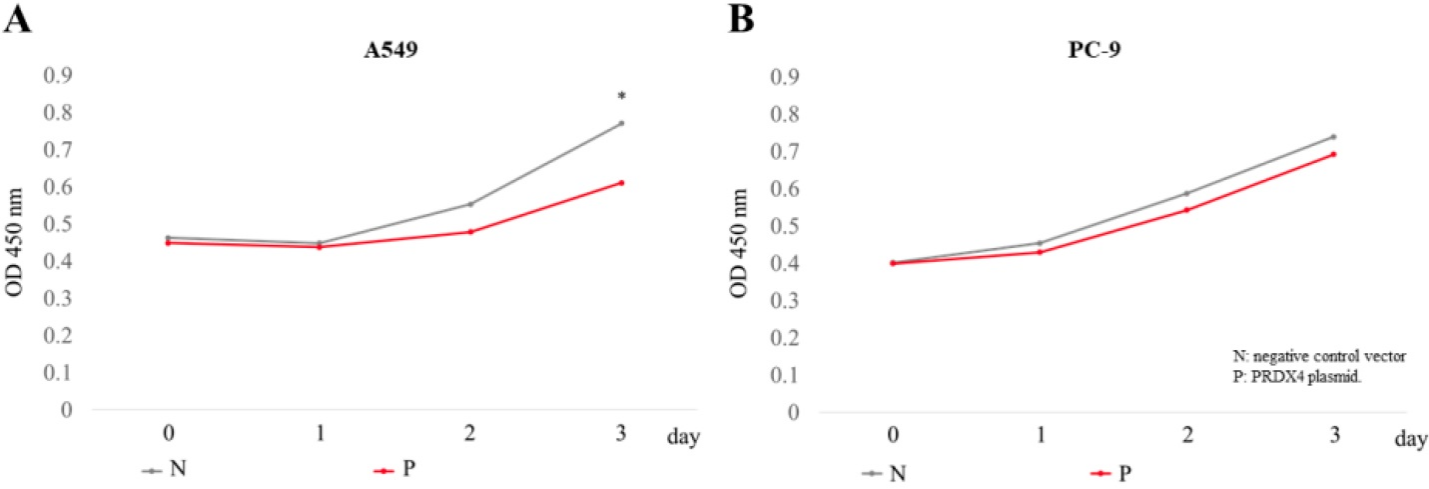
** The overexpression of PRDX4 inhibited cell proliferation.** The proliferation of A549 **(A)** and PC-9 **(B)** cells was analyzed using a cck-8 kit, 3 days after the transfection of PRDX4 plasmid DNAs or negative vectors. N, negative control vector; P, PRDX4 plasmid DNA. *p<0.05

Since it is well known that EGFR mutations usually lead to the abnormal expression of itself and promotes cellular proliferation in lung cancer, we investigated the roles of PRDX4 in the proliferation of two human LUAD cell lines, A549 cells (EGFR wild-type) and PC-9 cells (EGFR mutant). A significant increase in the expression of PRDX4 mRNA and protein was observed 3 days after transfection with PRDX4 plasmid DNA in both A549 and PC-9 cells (Fig. 3). Viability assays performed using a Cell Counting Kit-8 showed that the proliferation of A549 cells (EGFR wild-type) was significantly suppressed after the upregulation of the PRDX4 expression (Fig. 4A), but the overexpression of PRDX4 did not affect the proliferation of PC-9 cells (EGFR mutant) (Fig. 4B), indicating that EGFR mutation may attenuate the inhibitory role of PRDX4 in human LUAD cells.

## Discussion

The analysis of patients with stage I LUAD revealed that the EGFR mutant group included a larger number of patients with high PRDX4 expression levels than the EGFR wild-type group, indicating that there is a close relationship between the expression of PRDX4 and the EGFR mutation status. In addition, a Kaplan-Meier analysis showed that patients with EGFR mutations and high PRDX4 expression levels had significantly improved DFS in comparison to other patients, while the DFS of patients with EGFR wild-type and low PRDX4 expression levels was significantly reduced in comparison to other patients, suggesting that the combination of the PRDX4 expression and the EGFR mutation status may be an independent marker for postoperative recurrence in stage I LUAD patients.

EGFR belongs to the transmembrane receptor Tyr kinase family, which is triggered by ligand binding and the induction of EGFR homo-dimerization or hetero-dimerization 37 and regulates cell proliferation, migration, and survival 38. The aberrant expression of the EGFR via gene amplification, mutation, or the overexpression of protein results in tumorigenesis, due to dysregulation of the EGFR-mediated signaling pathways, especially in lung cancer 39, 40. Generally, the mutation of EGFR will lead to its overexpression 41, which could lead to excessive growth stimulation with the excess production of ROS 42. Thus, in the present study, oxidative stress and an altered redox environment probably induced the expression of more antioxidants, including PRDX4, in order to promote cell survival. Several EGFR Tyr kinase inhibitors (TKIs) have been used as anticancer agents in the treatment of NSCLC in patients with EGFR mutations 43, and EGFR-TKIs show an positive therapeutic effect on EGFR-mutated NSCLC, recurrence and resistance to EGFR TKIs in LUAD patients after curative surgery remains a significant problem and limits the application of EGFR TKIs in NSCLC treatment 44, which leads to a poor prognosis in lung cancer patients. Thus, a better understanding of EGFR-regulated signaling pathways or other molecular mechanisms related to EGFR signaling is likely to have important clinical significance in cancer therapy for lung cancer patients. We summarized the hypothesized interactions between PRDX4 and EGFR in Supplementary Fig. 1.

In previous studies, we reported that tumor tissues of hepatocellular carcinoma and LUAD with high PRDX4 expression levels were significantly larger in size in comparison to those with low PRDX4 expression levels 21, 23, which was closely associated with the prognosis of these patients, suggesting that PRDX4 plays an important role in the proliferation of carcinoma cells. Indeed, in this study, our results showed that the overexpression of PRDX4 suppressed the rate of A549 cell proliferation, which was in line with the findings from our previous clinical study. Interestingly, however, the suppressive role of PRDX4 in cell proliferation was not observed in PC-9 cells (with EGFR mutation). The relatively higher ROS levels are suggested to be related to the proliferation of cancer cells 45. Thus, it is easy to understand that the overexpression of PRDX4 may downregulate the intracellular ROS levels, which led to the inhibitory effects on the proliferation in A549 cells. However, EGFR-related molecular mechanisms may play more important roles in the processes of cell proliferation of PC-9 cells. At present, the precise mechanism underlying the inhibition of the suppressive roles of PRDX4 in the cellular proliferation of LUAD cells with EGFR mutations remains unclear. Further laboratory experiments are necessary to understand these findings.

The present study was associated with some limitations. Although we suggest that the number of human specimens was reasonable in this study, some important clinicopathological parameters related to DFS could not reach to statistical significance. Besides, all of the sample subjects resided relatively near to the single institution and had similar living habits with similar climatic conditions; thus, we could not avoid making a partial conclusion thoroughly. In addition, the *in vitro* analysis of cell proliferation only included one EGFR mutant cell line and the EGFR wild-type cell line.

## Conclusion

In summary, the present study revealed—for the first time—a specific relationship between the expression PRDX4 and the EGFR mutation status, and further found that the combination of high-PRDX4 and EGFR mutation was closely associated with an improved prognosis whereas low-PRDX4 and EGFR wild-type was associated with worse DFS, suggesting that the combination of the PRDX4 expression and EGFR mutation status may be a novel prognostic biomarker for patients with stage I LUAD who have undergone surgery. Moreover, EGFR mutations may inhibit the suppressive role of PRDX4 in the proliferation of LUAD cells. Further laboratory experiments are needed to confirm the precise mechanism.

## Supplementary Material

Supplementary figures and tables.

## Figures and Tables

**Table 1:** The clinicopathological characteristics of the patients

Characteristics	Patients (n=127)
Age (years)	
Average	68
Median	69
Range	34-84
>60	106
≤60	21
Sex	
Male	63
Female	64
Brinkman index (BI)	
≥400	47
<400	80
Tumor differentiation	
Well	72
Moderate	48
Poor	7
Tumor size (mm)	
Average	22
Median	21
Range	6-50
pl	
(+)	22
(-)	105
ly	
(+)	46
(-)	80
v	
(+)	43
(-)	83

**Table 2:** The relationship between the EGFR mutation status and the expression of PRDX4

	High-PRDX4 (n=69)	Low-PRDX4 (n=58)	P
**EGFR mutant (n=75)**	53	22	**<0.0001**
**EGFR wild-type (n=52)**	16	36	

## Abbreviations

LUAD: lung adenocarcinoma
PRDX4: peroxiredoxin 4
NSCLC: non-small cell lung cancer
TNM: tumor-node-metastasis
ROS: reactive oxygen species
DFS: disease-free survival
ROC: receiver operating characteristic
