# Supplementary material for: The impact of PRDX4 and the EGFR mutation status on cellular proliferation in lung adenocarcinoma
Source: Int J Med Sci. 2019 Aug 14;16(9):1199–206. doi: 10.7150/ijms.36071 (PMC6775271; doi:10.7150/ijms.36071)

Supplementary Table 1. The clinicopathological characteristics of the EGFR wild-type and low-PRDX4 group and the other patients

	**EGFR wild-type + low-PRDX4 (n=36)**	**Others (n=91)**	**P**
**Age**			
＞60 years	26	80	0.0601
≤60 years	10	11	
**Sex**			
Male	27	36	0.0007
Female	9	55	
**Brinkman index (BI)**			
≥400	23	24	0.0002
＜400	13	67	
**Tumor size**			
＞2 cm	15	52	0.1684
≤2 cm	21	39	
**pl**			
(+)	10	12	0.0895
(-)	26	79	
**ly**			
(+)	13	33	1.0000
(-)	23	57	
**v**			
(+)	15	28	0.3571
(-)	21	62	

Supplementary Table 2. The clinicopathological characteristics of the EGFR mutant and high-PRDX4 group and the other patients

	**EGFR mutant + high-PRDX4 (n=53)**	**Others (n=74)**	**P**
**Age**			
＞60 years	46	60	0.5404
⩽60 years	7	14	
**Sex**			
Male	22	41	0.1724
Female	31	33	
**Brinkman index (BI)**			
⩾400	12	35	0.0080
＜400	41	39	
**Tumor size**			
＞2 cm	28	39	1.0000
⩽2 cm	25	35	
**pl**			
(+)	5	17	0.0801
(-)	48	57	
**ly**			
(+)	19	27	1.0000
(-)	34	46	
**v**			
(+)	15	28	0.3247
(-)	38	45	

Supplementary Figure 1. A schematic illustration of the suppressive role of PRDX against growth stimuli. EGFR, epidermal growth factor receptor; PTP, phosphotyrosine phosphatase; ROS, reactive oxygen species; PRDX4, peroxiredoxin 4.

Supplementary Figure 1

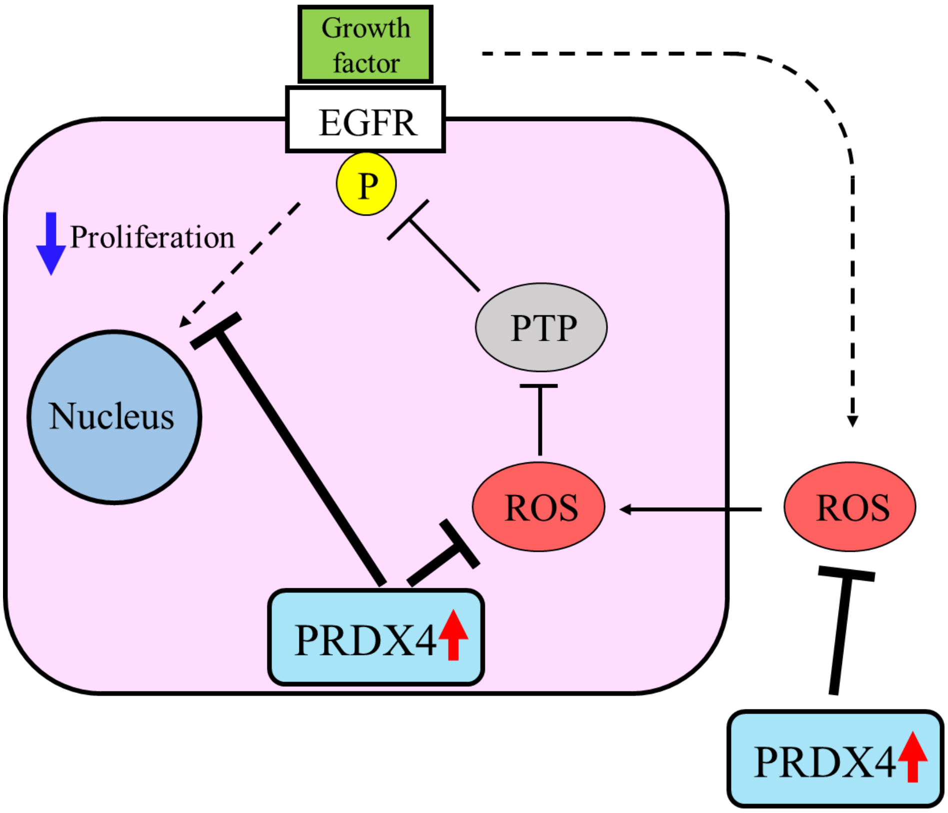

Supplement: Supplementary file 1 — Supplementary figures and tables. [file ijmsv16p1199s1.pdf]
